# *Drosophila*: a Tale of regeneration with MYC

**DOI:** 10.3389/fcell.2024.1429322

**Published:** 2024-07-23

**Authors:** Florenci Serras, Paola Bellosta

**Affiliations:** ^1^ Department of Genetics, Microbiology and Statistics, Faculty of Biology, and Institute of Biomedicine of the University of Barcelona (IBUB), Barcelona, Spain; ^2^ Department of Cellular, Computational and Integrative Biology (CiBiO), University of Trento, Trento, Italy; ^3^ Department of Medicine, NYU Langone Medical Center, New York, NY, United States

**Keywords:** MYC, regeneration, imaginal discs, epithelial cells, gut, neurons and glia, *Drosophila*

## Abstract

Regeneration is vital for many organisms, enabling them to repair injuries and adapt to environmental changes. The mechanisms underlying regeneration are complex and involve coordinated events at the cellular and molecular levels. Moreover, while some species exhibit remarkable regenerative capabilities, others, like mammals, have limited regenerative potential. Central to this process is the regulation of gene expression, and among the numerous genes involved, MYC emerges as a regulator of relevant processes during regeneration with roles conserved in several species, including *Drosophila*. This mini-review aims to provide valuable insights into the regeneration process in flies, focusing on significant organs where the role of MYC has been identified: from the imaginal discs, where MYC regulates cell growth, structure, and proliferation, to the gut, where it maintains the balance between renewal and differentiation of stem cells, and the central nervous system, where it influences the activities of neural stem cells and the interaction between glia and neuronal cells. By emphasizing the molecular mechanisms regulated by MYC, its significance in controlling regeneration mechanisms, and its conserved role in flies, we aim to offer valuable insights into the utility of *Drosophila* as a model for studying regeneration. Moreover, unraveling MYC’s function in *Drosophila* during regeneration may help translate findings into the mechanisms underlying human tissue repair.

## 1 Regeneration

The ability to regenerate and restore lost body parts after injury reflects key physiological pathways governed by developmental processes; regeneration capacity is widespread in animals and, in some species, has been lost during evolution, contributing to the variations in regenerative capacities across species (Losner et al., 2021). While remarkable abilities are observed in cnidarians, crustaceans, salamanders, and certain vertebrates, humans have limited regenerative potential (Wells and Watt, 2018), underscoring the need to understand molecular mechanisms of tissue and organ development for regenerative medicine.

Animal regeneration is categorized into five types: 1) structural regeneration, seen in the distal regrowth of appendages in vertebrates and arthropods; 2) organ regeneration, where damaged organs restore their mass; 3) tissue regeneration, responding to damaged epithelial or epidermis; 4) whole-body regeneration, involving the regrowth of an organism’s central axis; and 5) cellular regeneration, such as the regrowth of severed nerve axons (Bely and Nyberg, 2010). Regeneration, depending on tissue and damage types, involves distinct steps, including wound healing, the formation of a proliferative blastema, cellular differentiation, and tissue patterning. The blastema, comprised of progenitor cells responsible for the regeneration process, is formed temporarily at the injury site and undergoes morphogenesis through cell migration and proliferation to regenerate the missing organ (King and Newmark, 2012; Slack, 2017). Additionally, immune cells at the injury site play a crucial role in debris clearance and secretion of signaling molecules, initiating specific cellular proliferation and differentiation processes necessary for thriving tissue regeneration (Julier et al., 2017). Despite the progress made in understanding tissue regeneration, identifying novel signaling pathways that govern reprogramming mechanisms remains a significant challenge. Consequently, simple animal models are indispensable for gaining a deeper understanding of these intricate processes.

Although *Drosophila* does not possess the extensive regenerative abilities of some other species, its advanced genetic technology, previously used to uncover the complex genetic networks governing development, framework, which connects body parts and identity genes (such as the Hox genes), as well as pattern formation components (like Hedgehog, Decapentaplegic (Dpp), and Wingless (Wg) analogous to vertebrate Wnt), can now be utilized to investigate the molecular basis of regeneration (Fox et al., 2020). Here, we review the role of MYC in regeneration models such as wing imaginal discs, gut, and neuronal-glia cells, where processes like cell growth, division, and apoptosis may depend critically on MYC’s function.

## 2 *Drosophila* MYC

The *MYC/MAX/MAD* network in *Drosophila* stands out for its lack of redundancy, as the *Drosophila* genome contains a single gene for each component (Gallant, 2006). Despite being only 26% identical to its human counterpart, the *Drosophila* MYC protein shares highly conserved functional domains such as Box I and II, the degron sequences, and the basic-helix-loop-helix leucine zipper (bHLH/LZ) domain, to mediate MYC: MAX heterodimers that bind the E-box sequences on target genes (Orian et al., 2003; Hulf et al., 2005). The discovery that *MYC* mutants, also called *diminutive*, are composed of smaller cells (Johnston et al., 1999) paved the way for genetic experiments that revealed MYC’s role in controlling growth and ribosomal biogenesis. The similarity in phenotypes between *MYC* mutants and those of the *insulin* (*InR/IRS/chico*) (Bohni et al., 1999) and Target of Rapamycin (*TOR/S6K*) (Montagne et al., 1999) pathways has contributed to unveiling how growth pathways influence MYC activity in flies (Bellosta and Gallant, 2010; Parisi et al., 2011). These studies revealed the control of MYC protein stability by growth factors signaling through the phosphorylation of conserved domains (degrons) by Ras-ERK/MAPK and GSK3ß kinases, confirming this pathway of MYC protein degradation in flies (Galletti et al., 2009; Schwinkendorf and Gallant, 2009). Furthermore, MYC levels increase during starvation in the fat body, a metabolic tissue that parallels the function of vertebrate adipose tissue and the liver (Teleman et al., 2008; Parisi et al., 2013). Indeed, we showed that MYC increases metabolic processes like glycolysis and glutaminolysis during nutrient starvation (Parisi et al., 2013; de la Cova et al., 2014) and promotes the catabolic process autophagy in the fat cells, leading to survival (Nagy et al., 2013; Paiardi et al., 2017).

MYC’s control over ribosome biogenesis is highlighted by its coordination of RNA polymerases I, II, and III activities. MYC facilitates the recruitment of RNA polymerase I to rDNA, ensuring proper rRNA synthesis with the transcription of ribosomal proteins (Destefanis et al., 2020). MYC’s role in regulating ribosomal biogenesis led to the discovery of its role in cell competition; a physiological process initially observed in flies heterozygous for the *Minute* ribosomal proteins (Morata and Ripoll, 1975). In this process, cells with higher MYC levels outcompete unfit neighboring cells (with lower MYC), leading to their apoptosis (de la Cova et al., 2004; Moreno and Basler, 2004). This property of MYC was later demonstrated in the development of vertebrates (Claveria et al., 2013; Ellis et al., 2019), and it may underscore a role for MYC in mechanisms of tissue repair and regeneration across diverse organisms (Gogna et al., 2015; Yusupova and Fuchs, 2023).

## 3 Organ-specific regeneration: the wing imaginal discs, gut, and neural cells, three models to study regeneration

### 3.1 Wing imaginal discs

**FIGURE 1:**
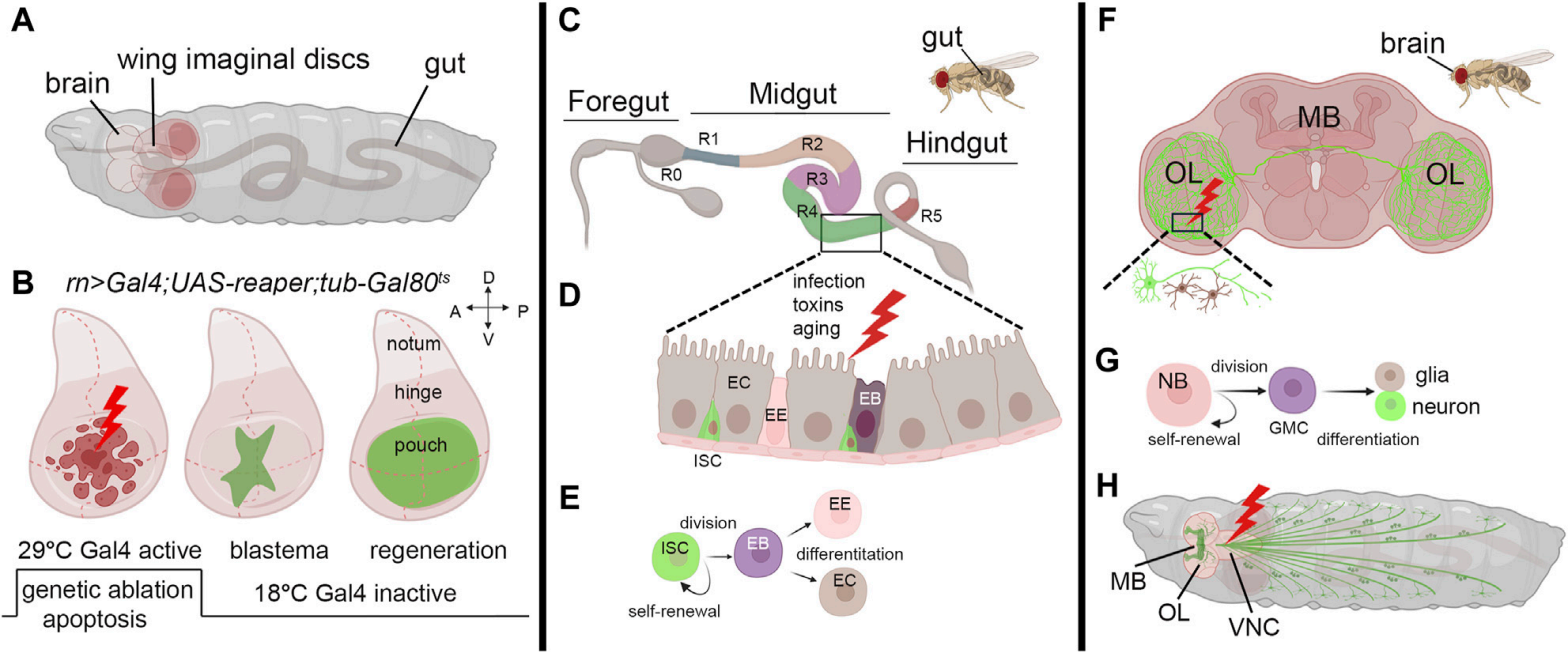
Models to study regeneration. **(A)** Schematic view of third instar larvae indicating the brain, wing imaginal discs, and the gut. **(B)** Third instar wing imaginal discs in which apoptosis is induced in the pouch using a specific Gal4-promoter. (Left) The induction of the apoptotic gene occurs through a controlled temperature switch. At 18°C, Gal80 binds to Gal4, repressing its activity and preventing its expression. However, when the temperature is switched to 29°C, Gal80 expression is suppressed, releasing Gal4 from inhibition and initiating the expression of the apoptotic gene and cell death (Hariharan and Serras, 2017). (Middle) After a few hours, animals are switched to the permissive temperature of 18°C to block apoptosis, allowing regeneration to occur with the formation of the blastema (green) that expands until a fully recovered pouch is obtained (Right). **(C)** Representation of the adult gut with the zone that characterizes its function (R0-5) (Buchon et al., 2013). **(D)** Model of the midgut epithelium where regeneration occurs upon injury. Cells are color-coded as in panel **(E)**, where the stem-cell niche is represented: ISC: Intestinal Stem Cell, EB: Enteroblast, EE: Enteroendocrine cell, EC: Enterocyte. **(F)** Schematic representation of the adult brain indicating the most common structures MB: mushroom body, OL: Optical Lobe. The inset represents a site of injury with neuron and glial cells represented in green and brown. **(G)** Representation of neuroblasts division. Neuroblasts (NB) divide asymmetrically, generating a ganglion mother cell (GMC), which then divides to produce a postmitotic neuron or glial cell (Homem and Knoblich, 2012). **(H)** Schematic representation of a third instar larva indicating the mushroom body (MB), the Optical lobe (OL) and the neurons (green). In red is a common site for injury in the Ventral Neural Cord (VNC). The figure was created using BioRender Premium, license (XV26VCD8GB), and further refined using Adobe Photoshop for its final appearance.

Imaginal discs in *Drosophila* larvae are sac-like structures of epithelial tissue (Figure 1A) and they are the precursors of adult organs. Due to their accessibility and the availability of a wide range of genetic tools, imaginal discs have become, in the last decade, an invaluable tissue for studying regeneration. They also provide an excellent platform for analyzing evolutionarily conserved pathways identified in the regeneration (Hariharan and Serras, 2017; Fox et al., 2020). Early studies on regeneration demonstrated that when imaginal wing discs were cut into small pieces and transplanted into either adult female abdomen, which served as natural culture chambers, or young larvae, they regenerated to their correct size and shape (Bergantinos et al., 2010b; Worley and Hariharan, 2022). This indicated the ability of the discs to resume proliferation and regenerate the missing part. These pioneering experiments demonstrated the regenerative potential of imaginal discs and unveiled their plasticity. In addition, fragments of discs cultured through prolonged transplantation cell-fate changes such as leg-to-wing, leading to the regeneration of alternative organs, in a phenomenon called transdetermination. This phenomenon demonstrates the capacity of *Drosophila* imaginal cells to be reprogrammed to various lineages (McClure and Schubiger, 2007). The refinement of surgical ablation of imaginal discs facilitated the exploration of regeneration during larval and pupal development. This technique revealed the critical role of cell division and the timing of ablation during development in shaping the regeneration timing (Diaz-Garcia and Baonza, 2013). More advanced technology was developed using genetic tools to induce apoptosis in specific domains of the disc and monitor tissue recovery, utilizing the binary UAS/Gal4 system (Brand and Perrimon, 1993). This widely used technique was adapted to study regeneration by temporally inducing the expression of apoptotic genes in the wing disc, regulated by the temperature-sensitive allele Gal80^ts^, an inhibitor of Gal4 (Figure 1B) (Smith-Bolton et al., 2009; Bergantinos et al., 2010a). Furthermore, the UAS/Gal4 system was combined with an engineered LexA-LexAop system, enabling precise temporal induction of cell death (Santabarbara-Ruiz et al., 2015). These methods allowed the identification of crucial genes involved in blastema formation, including Wg, a key regulator of regeneration in many species, and MYC (Smith-Bolton et al., 2009; Worley et al., 2012). Indeed, MYC was found to be upregulated in the proliferating cells surrounding the blastema, and its reduction partially impeded regeneration in the wing pouch (Smith-Bolton et al., 2009). Subsequent research demonstrated that MYC reduction, combined with *reaper* ablation, significantly hindered regeneration in the wing disc. Conversely, under the same conditions, MYC overexpression improved both the size and morphology of the adult wings, confirming its crucial role in the regeneration process (Harris et al., 2020). Additionally, MYC has been identified to regulate Yorkie (Yki), the unique *Drosophila* ortholog of YAP/TAZ, a component of the Hippo tumor suppressor pathway, in a feed-back mechanism that restrains the growth of the imaginal discs (Neto-Silva et al., 2010; Ziosi et al., 2010). In mammals, the Hippo-YAP/TAZ pathway regulates regeneration by controlling cell proliferation, apoptosis, and stem cell maintenance to ensure proper tissue growth and repair (Moya and Halder, 2019). Thus, MYC’s regulation of Yorkie (Yki) could be crucial for balancing cell proliferation and tissue growth in response to damage. This coordination is vital for developmental processes and organ growth, where MYC and the Hippo pathway are key players (de la Cova et al., 2004; Pan, 2007). Cells at the regeneration site stimulate proliferation through non-autonomous mechanisms such as apoptosis-induced proliferation (AiP), compensating for the apoptotic zones by triggering cell proliferation (Fogarty and Bergmann, 2017). The mechanisms controlling AiP are still under investigation; however, one hypothesis is that the release of ROS by the dying cells activates the ROS-sensitive kinase 1 (Ask1), expressed during regeneration, and its signal attenuated by Akt1/PKB/InR in living cells surrounding the blastema modulates moderate JNK/p38 signaling, which is crucial for controlling apoptosis in the regenerative response (Santabarbara-Ruiz et al., 2019; Esteban-Collado et al., 2021). Recent single-cell transcriptomics analysis of blastema from wing imaginal discs identified Ets21C, a transcription factor that controls patterning and organ development. This factor is induced by cell damage and is essential for the expression of genes crucial for regeneration (Worley et al., 2022). Interestingly, our RNA sequencing data reveals that both Ets21C and MYC are upregulated in wing disc cells undergoing apoptosis induced by proteotoxic stress (not published), suggesting that their expression may share components in the stress response pathways still to be investigated.

**FIGURE 2:**
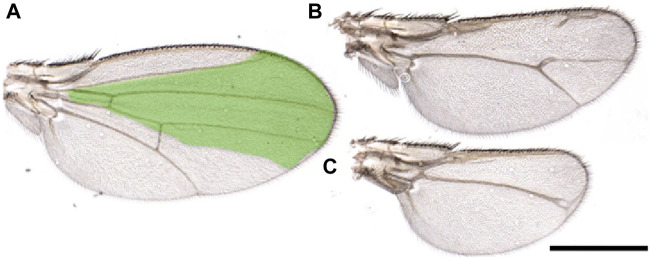
Starvation affects wing regeneration. **(A-C)** Wings from animals that underwent regeneration while subjected to amino acid starvation. Reaper was temporarily induced in the spalt domain (green), three days after egg laying in larvae of the genotype: *SpaltPE-Gal4/tub-Gal80* ^*ts*^ *; UAS-rpr*. Animals were kept in a starvation medium (PBS/20% sucrose) until eclosion. **(A)** Wings from flies not expressing *reaper*. **(B, C)** or in which r*eaper* was induced. These images highlight the morphological defects observed in the wings due to the incomplete regeneration process. Scale Bar 1 mm.

Finally, we would like to briefly address the critical role of the steroid hormone ecdysone during regeneration and its relation with MYC. Ecdysone controls cellular and specific pathways that regulate physiological organ growth and developmental timing (Andersen et al., 2013). Ecdysone is produced by the prothoracic gland (PG) at specific development times to regulate larval molting and metamorphosis (Edgar, 2006; Tennessen and Thummel, 2011). In the regeneration process, ecdysone levels determine the timing after which larvae terminate their window of regenerative potential by controlling the state of epithelial cell progenitors through regulating the transcription factors *chinmo* and *broad* (Narbonne-Reveau and Maurange, 2019; Karanja et al., 2022). Moreover, the release of ecdysone by the PG is indirectly controlled by the dying cells in the regenerating discs that secrete Dilp8, a peptide belonging to the insulin/relaxin-like growth factor family, which binds to the LGR3 receptor in the brain. This inhibits the release of ecdysone from the PG (Colombani et al., 2015; Vallejo et al., 2015) and slows down the development, allowing the damaged cells of the discs to complete their regeneration process (Blanco-Obregon et al., 2022; Karanja et al., 2022). Moreover, the physiological reduction of ecdysone at specific development points corresponds to an increase in MYC in the fat body (FB) (Delanoue et al., 2010). MYC in the FB favors the storage of nutrients (fat and sugars) and activates survival pathways such as autophagy to survive starvation (Parisi et al., 2013; Paiardi et al., 2017). It is known that regeneration in wing discs is affected by pathways regulated by nutrients (Esteban-Collado et al., 2021), and animals allowed to regenerate in starvation do not complete this process (Figure 2). The observation that animals in starvation have a reduced ability to regenerate suggests that non-autonomous signals from the FB are necessary to complete this process. Although MYC is upregulated in the FB of starved animals (Teleman et al., 2008; Parisi et al., 2013), the impaired regeneration observed under starvation conditions indicates that the upregulation of endogenous MYC activity in the FB is insufficient to sustain regeneration. Alternatively, starvation may prevent the storage of nutrients in the FB or hinder the production/secretion of factors necessary for regeneration.

### 3.2 Gut

Research on *Drosophila* gut regeneration offers valuable insights into repair mechanisms relevant to regenerative medicine, given the similarities in tissue composition, anatomy, and physiological functions with the human intestine. To investigate regeneration, various methods are employed to induce stress and cell damage, such as chemical exposure (e.g., Dextran Sulfate Disodium (DSS), bacterial infection, heat stress, oxidative stress (e.g., H_2_O_2_), and mechanical damage (Apidianakis and Rahme, 2011; Zhang and Edgar, 2022) *Drosophila* gut comprises an anterior, middle, and posterior hindgut (Figure 1C); however, regeneration primarily occurs in the midgut, where the Intestinal Stem Cells (ISCs) generate a niche initiated by Notch (Ohlstein and Spradling, 2007). These cells divide asymmetrically and give rise to a new ISC and an Enteroblast (EB) that will differentiate into Enterocytes (ECs) or Enteroendocrine cells (EE) in the absence of cell division (Figures 1D,E) (Mathur et al., 2010; Amcheslavsky et al., 2014). Wg is necessary to maintain ISCs self-renewal and is the balance between Notch and Wg signaling that controls the equilibrium between the proliferation and differentiation of ISCs (Zhang and Edgar, 2022). MYC plays a crucial role in mediating gut fitness both in ISCs and in ECs. MYC activity is essential for their differentiation and proliferation and acts downstream of stress-dependent and growth factor pathways such as JAK-STAT, Wg, Hippo, and EGFR (Ren et al., 2013). MYC is also crucial in maintaining gut health in response to different diet conditions. A nutrient-rich diet suppresses MYC in ECs, increasing cell death and gut permeability and shortening lifespan. Conversely, dietary restriction boosts MYC, enhancing EC fitness, gut integrity, and lifespan (Akagi et al., 2018). This may occur through MYC-inducing cell competition, which is crucial for maintaining the fitness of adult enterocytes (ECs), especially during dietary changes. Interestingly, this is similar to what was previously described in intestinal ISCs for *Minute* genes, many of which are MYC targets, where both ISC and differentiated *Minute*/+ cells were eliminated through cell competition to promote the proliferation and self-renewal of wild-type stem cells (Kolahgar et al., 2015). Recent evidence also reveals the role of MYC as a regulator of the amino acid transporter *arcus* (*acs*) in ECs (Socha et al., 2023). This signal is coordinated with the activation of the insulin pathway that favors aminoacidic absorption and ECs recovery after bacterial-mediated toxin damage, suggesting another active role for MYC in the gut to favor the regeneration of these cells.

### 3.3 Neuronal cells

*Drosophila*’s neural stem cells (NSCs), or neuroblasts, are pivotal for brain development. They exhibit remarkable plasticity, transitioning between quiescent and active states in response to environmental cues or injury. This dynamic regulation underscores their importance in maintaining brain homeostasis and promoting tissue repair. Neuroblasts (NB) play a crucial role in larval development, undergoing asymmetric division to generate neuroblasts and smaller ganglion mother cells (GMC). These GMCs divide further to produce post-mitotic neurons or glial cells (Figure 1G) (Homem and Knoblich, 2012; Otsuki and Brand, 2020). The neurons establish identities via proneural and selector genes, resulting in four classes (I-IV) of dendritic arborization (da) sensory neurons. Class IV-ddaC neurons, known for their intricate dendritic arbors sensitive to mechanical stimuli, serve as models for dendrite repair and the study of neurodevelopmental disorders (Grueber et al., 2007; Liu et al., 2023).

Methods for investigating neuronal regeneration during development include gently crushing the larval segmental nerve to maintain larval viability or employing laser ablation (Figure 1H). This approach involves labeling specific axon patterns using GFP expressed by neuronal-specific promoters, facilitating the visualization of cells during regeneration events throughout larval development (Pfeiffer et al., 2008). In adult flies, few models exist for studying neuronal regeneration. Experimental stab lesions to either the optic lobes (OL) or the central brain result in local neurogenesis days after injury (Figure 1F). This response was attributed to dormant neural progenitor cells (qNPs) activation (Moreno et al., 2015; Crocker et al., 2021). Glial cells respond to nervous system damage by increasing their number and changing morphology after neuronal cell death. This process is conserved across regions of the peripheral nervous system and involves Dpp and Hh signaling, with the JNK pathway contributing to glial migration (Velarde et al., 2021). Glial cells exhibit an immune response like microglia, expressing the phagocytic receptor draper (drpr), crucial for axon regeneration and debris clearance. While macrophages aid central nervous system (CNS) regeneration in vertebrates, their role in *Drosophila* neural injury remains unclear (Losada-Perez et al., 2021).

Recent discoveries highlight the crucial role of NSCs in maintaining and regenerating adult brain tissues (Li and Hidalgo, 2020). In contrast to adult mammals, *Drosophila* NSCs can be activated by different diets or exercises initiated by larval hatching. However, the mechanisms by which NSCs transition between quiescence and activation remain elusive (Ding et al., 2020). Brain injuries in adult flies are thought to trigger the recruitment of quiescent neural progenitors (qNPs) near the injury site, facilitated by damage-responsive neuroglial clusters (DNGCs). These clusters stimulate the proliferation of distant qNPs, thereby expanding the zone of stem cell activation through the reactivation of dormant qNPs (Moreno et al., 2015; Crocker et al., 2021). Since previous research has shown that a ubiquitous pulse of MYC promotes qNP division (Fernandez-Hernandez et al., 2013), it is possible that MYC could induce growth factors in qNPs through injury-induced secretion, allowing these cells to survive and proliferate. MYC has also emerged as a non-autonomous regulator of metabolism in retinal ganglion glial cells, where using a model of reprogrammed glial cells that activate PI3K and EGFR pathways (RGCPE), MYC activity was shown relevant for the regeneration of neurons by mediating pro-regeneration metabolic pathways in glia (Li et al., 2020), including the glia-neuron lactate shuttle essential for neuronal survival (Volkenhoff et al., 2015). This highlights its important role in inducing nonautonomous signals that control axon regeneration.

## 4 Discussion

Studying regeneration in *Drosophila* has unveiled complex cellular and molecular mechanisms guiding tissue repair and organ regeneration across species. Although tissues display differing regenerative abilities, common pathways and principles govern regeneration. The pivotal role of MYC emphasizes its importance in regulating fundamental conserved processes, connecting metabolism and growth, influencing cell competition, and highlighting regeneration’s complexity. Insights from *Drosophila* research hold potential for future advancements in regenerative medicine. Further exploring molecular mechanisms across organisms is fundamental to developing novel therapeutic strategies to enhance human tissue repair and organ regeneration.

## References

[B1] Akagi K. Wilson K. A. Katewa S. D. Ortega M. Simons J. Hilsabeck T. A. (2018). Dietary restriction improves intestinal cellular fitness to enhance gut barrier function and lifespan in *D. melanogaster* . PLoS Genet. 14, e1007777. 10.1371/journal.pgen.1007777 30383748 PMC6233930

[B2] Amcheslavsky A. Song W. Li Q. Nie Y. Bragatto I. Ferrandon D. (2014). Enteroendocrine cells support intestinal stem-cell-mediated homeostasis in Drosophila. Cell Rep. 9, 32–39. 10.1016/j.celrep.2014.08.052 25263551 PMC4198943

[B3] Andersen D. S. Colombani J. Leopold P. (2013). Coordination of organ growth: principles and outstanding questions from the world of insects. Trends Cell Biol. 23, 336–344. 10.1016/j.tcb.2013.03.005 23587490

[B4] Apidianakis Y. Rahme L. G. (2011). *Drosophila melanogaster* as a model for human intestinal infection and pathology. Dis. Model Mech. 4, 21–30. 10.1242/dmm.003970 21183483 PMC3014343

[B5] Bellosta P. Gallant P. (2010). Myc function in Drosophila. Genes Cancer 1, 542–546. 10.1177/1947601910377490 21072325 PMC2976539

[B6] Bely A. E. Nyberg K. G. (2010). Evolution of animal regeneration: re-emergence of a field. Trends Ecol. Evol. 25, 161–170. 10.1016/j.tree.2009.08.005 19800144

[B7] Bergantinos C. Corominas M. Serras F. (2010a). Cell death-induced regeneration in wing imaginal discs requires JNK signalling. Development 137, 1169–1179. 10.1242/dev.045559 20215351

[B8] Bergantinos C. Vilana X. Corominas M. Serras F. (2010b). Imaginal discs: renaissance of a model for regenerative biology. Bioessays 32, 207–217. 10.1002/bies.200900105 20127699

[B9] Blanco-Obregon D. El Marzkioui K. Brutscher F. Kapoor V. Valzania L. Andersen D. S. (2022). A Dilp8-dependent time window ensures tissue size adjustment in Drosophila. Nat. Commun. 13, 5629. 10.1038/s41467-022-33387-6 36163439 PMC9512784

[B10] Bohni R. Riesgo-Escovar J. Oldham S. Brogiolo W. Stocker H. Andruss B. F. (1999). Autonomous control of cell and organ size by CHICO, a Drosophila homolog of vertebrate IRS1-4. Cell 97, 865–875. 10.1016/s0092-8674(00)80799-0 10399915

[B11] Brand A. H. Perrimon N. (1993). Targeted gene expression as a means of altering cell fates and generating dominant phenotypes. Development 118, 401–415. 10.1242/dev.118.2.401 8223268

[B12] Buchon N. Osman D. David F. P. Fang H. Y. Boquete J. P. Deplancke B. (2013). Morphological and molecular characterization of adult midgut compartmentalization in Drosophila. Cell Rep. 3, 1725–1738. 10.1016/j.celrep.2013.04.001 23643535

[B13] Claveria C. Giovinazzo G. Sierra R. Torres M. (2013). Myc-driven endogenous cell competition in the early mammalian embryo. Nature 500, 39–44. 10.1038/nature12389 23842495

[B14] Colombani J. Andersen D. S. Boulan L. Boone E. Romero N. Virolle V. (2015). Drosophila Lgr3 couples organ growth with maturation and ensures developmental stability. Curr. Biol. 25, 2723–2729. 10.1016/j.cub.2015.09.020 26441350

[B15] Crocker K. L. Marischuk K. Rimkus S. A. Zhou H. Yin J. C. P. Boekhoff-Falk G. (2021). Neurogenesis in the adult Drosophila brain. Genetics 219, iyab092. 10.1093/genetics/iyab092 34117750 PMC8860384

[B16] De La Cova C. Abril M. Bellosta P. Gallant P. Johnston L. A. (2004). Drosophila myc regulates organ size by inducing cell competition. Cell 117, 107–116. 10.1016/s0092-8674(04)00214-4 15066286

[B17] De La Cova C. Senoo-Matsuda N. Ziosi M. Wu D. C. Bellosta P. Quinzii C. M. (2014). Supercompetitor status of Drosophila myc cells requires p53 as a fitness sensor to reprogram metabolism and promote viability. Cell Metab. 19, 470–483. 10.1016/j.cmet.2014.01.012 24561262 PMC3970267

[B18] Delanoue R. Slaidina M. Leopold P. (2010). The steroid hormone ecdysone controls systemic growth by repressing dMyc function in Drosophila fat cells. Dev. Cell 18, 1012–1021. 10.1016/j.devcel.2010.05.007 20627082

[B19] Destefanis F. Manara V. Bellosta P. (2020). Myc as a regulator of ribosome biogenesis and cell competition: a link to cancer. Int. J. Mol. Sci. 21, 4037. 10.3390/ijms21114037 32516899 PMC7312820

[B20] Diaz-Garcia S. Baonza A. (2013). Pattern reorganization occurs independently of cell division during Drosophila wing disc regeneration *in situ* . Proc. Natl. Acad. Sci. U.S.A. 110, 13032–13037. 10.1073/pnas.1220543110 23878228 PMC3740865

[B21] Ding W. Y. Huang J. Wang H. (2020). Waking up quiescent neural stem cells: molecular mechanisms and implications in neurodevelopmental disorders. PLoS Genet. 16, e1008653. 10.1371/journal.pgen.1008653 32324743 PMC7179833

[B22] Edgar B. A. (2006). How flies get their size: genetics meets physiology. Nat. Rev. Genet. 7, 907–916. 10.1038/nrg1989 17139322

[B23] Ellis S. J. Gomez N. C. Levorse J. Mertz A. F. Ge Y. Fuchs E. (2019). Distinct modes of cell competition shape mammalian tissue morphogenesis. Nature 569, 497–502. 10.1038/s41586-019-1199-y 31092920 PMC6638572

[B24] Esteban-Collado J. Corominas M. Serras F. (2021). Nutrition and PI3K/Akt signaling are required for p38-dependent regeneration. Development 148, dev197087. 10.1242/dev.197087 33913483

[B25] Fernandez-Hernandez I. Rhiner C. Moreno E. (2013). Adult neurogenesis in Drosophila. Cell Rep. 3, 1857–1865. 10.1016/j.celrep.2013.05.034 23791523

[B26] Fogarty C. E. Bergmann A. (2017). Killers creating new life: caspases drive apoptosis-induced proliferation in tissue repair and disease. Cell Death Differ. 24, 1390–1400. 10.1038/cdd.2017.47 28362431 PMC5520457

[B27] Fox D. T. Cohen E. Smith-Bolton R. (2020). Model systems for regeneration: Drosophila. Development 147, dev173781. 10.1242/dev.173781 32253254 PMC7157589

[B28] Gallant P. (2006). Myc/Max/Mad in invertebrates: the evolution of the Max network. Curr. Top. Microbiol. Immunol. 302, 235–253. 10.1007/3-540-32952-8_9 16620031

[B29] Galletti M. Riccardo S. Parisi F. Lora C. Saqcena M. K. Rivas L. (2009). Identification of domains responsible for ubiquitin-dependent degradation of dMyc by glycogen synthase kinase 3beta and casein kinase 1 kinases. Mol. Cell Biol. 29, 3424–3434. 10.1128/MCB.01535-08 19364825 PMC2698736

[B30] Gogna R. Shee K. Moreno E. (2015). Cell competition during growth and regeneration. Annu. Rev. Genet. 49, 697–718. 10.1146/annurev-genet-112414-055214 26631518

[B31] Grueber W. B. Ye B. Yang C. H. Younger S. Borden K. Jan L. Y. (2007). Projections of Drosophila multidendritic neurons in the central nervous system: links with peripheral dendrite morphology. Development 134, 55–64. 10.1242/dev.02666 17164414

[B32] Hariharan I. K. Serras F. (2017). Imaginal disc regeneration takes flight. Curr. Opin. Cell Biol. 48, 10–16. 10.1016/j.ceb.2017.03.005 28376317 PMC5591769

[B33] Harris R. E. Stinchfield M. J. Nystrom S. L. Mckay D. J. Hariharan I. K. (2020). Damage-responsive, maturity-silenced enhancers regulate multiple genes that direct regeneration in Drosophila. Elife 9, e58305. 10.7554/eLife.58305 32490812 PMC7299344

[B34] Homem C. C. Knoblich J. A. (2012). Drosophila neuroblasts: a model for stem cell biology. Development 139, 4297–4310. 10.1242/dev.080515 23132240

[B35] Hulf T. Bellosta P. Furrer M. Steiger D. Svensson D. Barbour A. (2005). Whole-genome analysis reveals a strong positional bias of conserved dMyc-dependent E-boxes. Mol. Cell Biol. 25, 3401–3410. 10.1128/MCB.25.9.3401-3410.2005 15831447 PMC1084277

[B36] Johnston L. A. Prober D. A. Edgar B. A. Eisenman R. N. Gallant P. (1999). Drosophila myc regulates cellular growth during development. Cell 98, 779–790. 10.1016/s0092-8674(00)81512-3 10499795 PMC10176494

[B37] Julier Z. Park A. J. Briquez P. S. Martino M. M. (2017). Promoting tissue regeneration by modulating the immune system. Acta Biomater. 53, 13–28. 10.1016/j.actbio.2017.01.056 28119112

[B38] Karanja F. Sahu S. Weintraub S. Bhandari R. Jaszczak R. Sitt J. (2022). Ecdysone exerts biphasic control of regenerative signaling, coordinating the completion of regeneration with developmental progression. Proc. Natl. Acad. Sci. U.S.A. 119, e2115017119. 10.1073/pnas.2115017119 35086929 PMC8812538

[B39] King R. S. Newmark P. A. (2012). The cell biology of regeneration. J. Cell Biol. 196, 553–562. 10.1083/jcb.201105099 22391035 PMC3307701

[B40] Kolahgar G. Suijkerbuijk S. J. Kucinski I. Poirier E. Z. Mansour S. Simons B. D. (2015). Cell competition modifies adult stem cell and tissue population dynamics in a JAK-STAT-dependent manner. Dev. Cell 34, 297–309. 10.1016/j.devcel.2015.06.010 26212135 PMC4537514

[B41] Li F. Sami A. Noristani H. N. Slattery K. Qiu J. Groves T. (2020). Glial metabolic rewiring promotes axon regeneration and functional recovery in the central nervous system. Cell Metab. 32, 767–785. 10.1016/j.cmet.2020.08.015 32941799 PMC7642184

[B42] Li G. Hidalgo A. (2020). Adult neurogenesis in the Drosophila brain: the evidence and the void. Int. J. Mol. Sci. 21, 6653. 10.3390/ijms21186653 32932867 PMC7554932

[B43] Liu X. Zhao Y. Zou W. (2023). Molecular mechanisms of neurite regeneration and repair: insights from *C. elegans* and Drosophila. Cell Regen. 12, 12. 10.1186/s13619-022-00155-2 37005942 PMC10067779

[B44] Losada-Perez M. Garcia-Guillen N. Casas-Tinto S. (2021). A novel injury paradigm in the central nervous system of adult Drosophila: molecular, cellular and functional aspects. Dis. Model Mech. 14, dmm044669. 10.1242/dmm.044669 34061177 PMC8214735

[B45] Losner J. Courtemanche K. Whited J. L. (2021). A cross-species analysis of systemic mediators of repair and complex tissue regeneration. NPJ Regen. Med. 6, 21. 10.1038/s41536-021-00130-6 33795702 PMC8016993

[B46] Mathur D. Bost A. Driver I. Ohlstein B. (2010). A transient niche regulates the specification of Drosophila intestinal stem cells. Science 327, 210–213. 10.1126/science.1181958 20056890 PMC2857772

[B47] Mcclure K. D. Schubiger G. (2007). Transdetermination: Drosophila imaginal disc cells exhibit stem cell-like potency. Int. J. Biochem. Cell Biol. 39, 1105–1118. 10.1016/j.biocel.2007.01.007 17317270 PMC2000801

[B48] Montagne J. Stewart M. J. Stocker H. Hafen E. Kozma S. C. Thomas G. (1999). Drosophila S6 kinase: a regulator of cell size. Science 285, 2126–2129. 10.1126/science.285.5436.2126 10497130

[B49] Morata G. Ripoll P. (1975). Minutes: mutants of drosophila autonomously affecting cell division rate. Dev. Biol. 42, 211–221. 10.1016/0012-1606(75)90330-9 1116643

[B50] Moreno E. Basler K. (2004). dMyc transforms cells into super-competitors. Cell 117, 117–129. 10.1016/s0092-8674(04)00262-4 15066287

[B51] Moreno E. Fernandez-Marrero Y. Meyer P. Rhiner C. (2015). Brain regeneration in Drosophila involves comparison of neuronal fitness. Curr. Biol. 25, 955–963. 10.1016/j.cub.2015.02.014 25754635 PMC4386028

[B52] Moya I. M. Halder G. (2019). Hippo-YAP/TAZ signalling in organ regeneration and regenerative medicine. Nat. Rev. Mol. Cell Biol. 20, 211–226. 10.1038/s41580-018-0086-y 30546055

[B53] Nagy P. Varga A. Pircs K. Hegedus K. Juhasz G. (2013). Myc-driven overgrowth requires unfolded protein response-mediated induction of autophagy and antioxidant responses in *Drosophila melanogaster* . PLoS Genet. 9, e1003664. 10.1371/journal.pgen.1003664 23950728 PMC3738540

[B54] Narbonne-Reveau K. Maurange C. (2019). Developmental regulation of regenerative potential in Drosophila by ecdysone through a bistable loop of ZBTB transcription factors. PLoS Biol. 17, e3000149. 10.1371/journal.pbio.3000149 30742616 PMC6386533

[B82] Neto-Silva R. M. De Beco S. Johnston L. A. (2010). Evidence for a growth-stabilizing regulatory feedback mechanism between Myc and Yorkie, the Drosophila homolog of Yap. Dev. Cell. 19, 507–520. 20951343 10.1016/j.devcel.2010.09.009 PMC2965774

[B55] Ohlstein B. Spradling A. (2007). Multipotent Drosophila intestinal stem cells specify daughter cell fates by differential notch signaling. Science 315, 988–992. 10.1126/science.1136606 17303754

[B56] Orian A. Van Steensel B. Delrow J. Bussemaker H. J. Li L. Sawado T. (2003). Genomic binding by the Drosophila Myc, Max, Mad/Mnt transcription factor network. Genes Dev. 17, 1101–1114. 10.1101/gad.1066903 12695332 PMC196053

[B57] Otsuki L. Brand A. H. (2020). Quiescent neural stem cells for brain repair and regeneration: lessons from model systems. Trends Neurosci. 43, 213–226. 10.1016/j.tins.2020.02.002 32209453

[B58] Paiardi C. Mirzoyan Z. Zola S. Parisi F. Vingiani A. Pasini M. E. (2017). The stearoyl-CoA desaturase-1 (Desat1) in Drosophila cooperated with myc to induce autophagy and growth, a potential new link to tumor survival. Genes (Basel) 8, 131. 10.3390/genes8050131 28452935 PMC5448005

[B59] Pan D. (2007). Hippo signaling in organ size control. Genes Dev. 21, 886–897. 10.1101/gad.1536007 17437995

[B60] Parisi F. Riccardo S. Daniel M. Saqcena M. Kundu N. Pession A. (2011). Drosophila insulin and target of rapamycin (TOR) pathways regulate GSK3 beta activity to control Myc stability and determine Myc expression *in vivo* . BMC Biol. 9, 65. 10.1186/1741-7007-9-65 21951762 PMC3235970

[B61] Parisi F. Riccardo S. Zola S. Lora C. Grifoni D. Brown L. M. (2013). dMyc expression in the fat body affects DILP2 release and increases the expression of the fat desaturase Desat1 resulting in organismal growth. Dev. Biol. 379, 64–75. 10.1016/j.ydbio.2013.04.008 23608455 PMC3712331

[B62] Pfeiffer B. D. Jenett A. Hammonds A. S. Ngo T. T. Misra S. Murphy C. (2008). Tools for neuroanatomy and neurogenetics in Drosophila. Proc. Natl. Acad. Sci. U. S. A. 105, 9715–9720. 10.1073/pnas.0803697105 18621688 PMC2447866

[B63] Ren F. Shi Q. Chen Y. Jiang A. Ip Y. T. Jiang H. (2013). Drosophila Myc integrates multiple signaling pathways to regulate intestinal stem cell proliferation during midgut regeneration. Cell Res. 23, 1133–1146. 10.1038/cr.2013.101 23896988 PMC3760623

[B64] Santabarbara-Ruiz P. Esteban-Collado J. Perez L. Viola G. Abril J. F. Milan M. (2019). Ask1 and Akt act synergistically to promote ROS-dependent regeneration in Drosophila. PLoS Genet. 15, e1007926. 10.1371/journal.pgen.1007926 30677014 PMC6363233

[B65] Santabarbara-Ruiz P. Lopez-Santillan M. Martinez-Rodriguez I. Binagui-Casas A. Perez L. Milan M. (2015). ROS-induced JNK and p38 signaling is required for unpaired cytokine activation during Drosophila regeneration. PLoS Genet. 11, e1005595. 10.1371/journal.pgen.1005595 26496642 PMC4619769

[B66] Schwinkendorf D. Gallant P. (2009). The conserved Myc box 2 and Myc box 3 regions are important, but not essential, for Myc function *in vivo* . Gene 436, 90–100. 10.1016/j.gene.2009.02.009 19248823

[B67] Slack J. M. (2017). Animal regeneration: ancestral character or evolutionary novelty? EMBO Rep. 18, 1497–1508. 10.15252/embr.201643795 28747491 PMC5579372

[B68] Smith-Bolton R. K. Worley M. I. Kanda H. Hariharan I. K. (2009). Regenerative growth in Drosophila imaginal discs is regulated by Wingless and Myc. Dev. Cell 16, 797–809. 10.1016/j.devcel.2009.04.015 19531351 PMC2705171

[B69] Socha C. Pais I. S. Lee K. Z. Liu J. Liegeois S. Lestradet M. (2023). Fast drosophila enterocyte regrowth after infection involves a reverse metabolic flux driven by an amino acid transporter. iScience 26, 107490. 10.1016/j.isci.2023.107490 37636057 PMC10448536

[B70] Teleman A. A. Hietakangas V. Sayadian A. C. Cohen S. M. (2008). Nutritional control of protein biosynthetic capacity by insulin via Myc in Drosophila. Cell Metab. 7, 21–32. 10.1016/j.cmet.2007.11.010 18177722

[B71] Tennessen J. M. Thummel C. S. (2011). Coordinating growth and maturation - insights from Drosophila. Curr. Biol. 21, R750–R757. 10.1016/j.cub.2011.06.033 21959165 PMC4353487

[B72] Vallejo D. M. Juarez-Carreno S. Bolivar J. Morante J. Dominguez M. (2015). A brain circuit that synchronizes growth and maturation revealed through Dilp8 binding to Lgr3. Science 350, aac6767. 10.1126/science.aac6767 26429885

[B73] Velarde S. B. Quevedo A. Estella C. Baonza A. (2021). Dpp and Hedgehog promote the glial response to neuronal apoptosis in the developing Drosophila visual system. PLoS Biol. 19, e3001367. 10.1371/journal.pbio.3001367 34379617 PMC8396793

[B74] Volkenhoff A. Weiler A. Letzel M. Stehling M. Klambt C. Schirmeier S. (2015). Glial glycolysis is essential for neuronal survival in Drosophila. Cell Metab. 22, 437–447. 10.1016/j.cmet.2015.07.006 26235423

[B75] Wells J. M. Watt F. M. (2018). Diverse mechanisms for endogenous regeneration and repair in mammalian organs. Nature 557, 322–328. 10.1038/s41586-018-0073-7 29769669

[B76] Worley M. I. Everetts N. J. Yasutomi R. Chang R. J. Saretha S. Yosef N. (2022). Ets21C sustains a pro-regenerative transcriptional program in blastema cells of Drosophila imaginal discs. Curr. Biol. 32, 3350–3364.e6. 10.1016/j.cub.2022.06.040 35820420 PMC9387119

[B77] Worley M. I. Hariharan I. K. (2022). Imaginal disc regeneration: something old, something new. Cold Spring Harb. Perspect. Biol. 14, a040733. 10.1101/cshperspect.a040733 34872971 PMC9620854

[B78] Worley M. I. Setiawan L. Hariharan I. K. (2012). Regeneration and transdetermination in Drosophila imaginal discs. Annu. Rev. Genet. 46, 289–310. 10.1146/annurev-genet-110711-155637 22934642

[B79] Yusupova M. Fuchs Y. (2023). To not love thy neighbor: mechanisms of cell competition in stem cells and beyond. Cell Death Differ. 30, 979–991. 10.1038/s41418-023-01114-3 36813919 PMC10070350

[B80] Zhang P. Edgar B. A. (2022). Insect gut regeneration. Cold Spring Harb. Perspect. Biol. 14, a040915. 10.1101/cshperspect.a040915 34312250 PMC8805648

[B83] Ziosi M. Baena-Lopez L. A. Grifoni D Froldi F. Pession A. Garoia F. (2010). dMyc functions downstream of Yorkie to promote the supercompetitive behavior of hippo pathway mutant cells. PLoS Genet. 6. 10.1371/journal.pgen.1001140 PMC2944792 20885789

